# Exploring a Potential Avenue for Beekeeping in Ireland: Safeguarding Locally Adapted Honeybees for Breeding Varroa-Resistant Lines

**DOI:** 10.3390/insects14100827

**Published:** 2023-10-20

**Authors:** Stephen Smith, Arrigo Moro, Grace P. McCormack

**Affiliations:** Galway Honey Bee Research Centre, Earth and Life Sciences, School of Natural Sciences, University of Galway, University Rd., H91 TK33 Galway, Ireland

**Keywords:** sustainable beekeeping, *Varroa destructor*, Varroa resistance, honey bee breeding, *Apis mellifera mellifera*

## Abstract

**Simple Summary:**

*Varroa destructor*, a parasitic mite, has significantly impacted Irish beekeeping, causing alarming colony losses and threatening the native honeybee subspecies, *Apis mellifera mellifera*. While Irish beekeepers are crucial for controlling this parasite, they lack supportive infrastructure. This creates a unique opening for national programmes promoting sustainable beekeeping practices for varroa control. Reports indicate that an increasing number of local beekeepers are successfully managing colonies without treatments, suggesting potential for varroa-resistant stock development through selective breeding. Our survey examined beekeepers’ views on sustainable practices and varroa resistance. The results underline Irish beekeepers’ hobbyist nature and their preference for the native honey bee subspecies. Some control varroa without treatment, achieving comparable survival rates. Most prefer varroa-resistant lines of native origin, with little openness to non-Irish strains. A strong willingness to engage in a national breeding programme appears evident. These insights offer Ireland an opportunity to establish a large-scale sustainable beekeeping strategy for safeguarding native honeybees and local biodiversity.

**Abstract:**

Beekeeping in Ireland has been strongly impacted by the parasitic mite *Varroa destructor*, whose introduction caused alarming honeybee colony losses. If unmitigated, these losses could lead to the disappearance of the native honeybee subspecies, *Apis mellifera mellifera*, with severe consequences for local biodiversity. Although beekeepers play a pivotal role in mitigating this crisis, beekeeping in Ireland is less intensive compared to other European regions, lacking significant infrastructure or support. These circumstances offer a unique opportunity for the development of national programmes that promote sustainable beekeeping practices for varroa control. Notably, local accounts highlight an increasing number of beekeepers successfully managing colonies in the absence of treatments, indicating a potential avenue for developing varroa-resistant stocks through selection of local colonies. Through a survey, we explored beekeeper’s opinions and attitudes towards future national projects focused on the development of sustainable beekeeping practices and selection for varroa resistance. The findings confirm the hobbyist nature of Irish beekeepers and their preference for the native honey bee. Some beekeepers were reported to be effectively controlling varroa without treatment, yielding comparable survivals to those using treatments. The majority expressed preference towards a varroa-resistant line if it were of native origin; a few were open to importing non-Irish lines. Overall, a strong willingness to participate in a national breeding programme was expressed. These findings highlight a prime opportunity for Ireland to establish a community-driven strategy based on sustainable beekeeping practices for safeguarding native honeybees and local biodiversity.

## 1. Introduction

The advent of modern beekeeping has had an enormous impact on the biology, evolution, and, ultimately, the fate of the Western honey bee, *Apis mellifera* [1]. The incremental professionalisation of this sector during the last century caused major changes, such as the spread of novel parasites throughout the world and the introduction of Western honey bee subspecies to non-native areas [2,3]. Such events then led to unsustainable managed-colony losses within the Northern Hemisphere, as well as a dramatic decrease in the species’ genetic diversity [4,5]. As such, nowadays, the beekeepers’ role has increased in its ecological significance, and this profession is called upon to play a fundamental role in the protection of honey bees’ health and diversity worldwide, including the adoption of more sustainable practices (e.g., [6,7]).

Beekeeping in Ireland is less intensive than in other European countries, with most beekeepers thought to be hobbyists rather than commercial [8]. While no official national register exists for beekeepers in Ireland, their number could be inferred from the number of registrants to the Irish beekeeping associations. The largest beekeeping organisation is the Federation of Irish Beekeepers’ Associations clg (FIBKA), currently with approximately 3000 members. The second-largest association is the Irish Beekeepers Association clg (IBA), with approximately 1200 members. Both are composed of a number of local associations with varying numbers of members. Furthermore, there exists a third national network, the Native Irish Honey Bee Society (NIHBS, [9]), which was established in 2012 with the aim of protecting and conserving the native honey bee subspecies, the dark honey bee, *A. m. mellifera*. This group is currently constituted of >1000 members composed of a mixture of FIBKA and IBA members and other individuals. Lastly, there are also many additional beekeepers (some known to the authors) who have not joined any association and who have also not registered their colonies with the Irish Department of Agriculture, Fisheries and the Marine.

As in many European regions, the native subspecies of *A. mellifera* in Ireland, *A. m. mellifera*, has come under significant threat [10]. While anecdotal reports indicate that honey bee colonies in Ireland were never very dense in number, it is considered that the population plummeted after *Varroa destructor*, an invasive parasite and the current biggest threat to honey bee health worldwide [3], was introduced to the island in 1998 [11]. If not controlled, this parasite can lead to the death of a non-adapted colony within two years [12]. As such, beekeepers started to apply chemical treatments to control its infestation and mitigate losses. However, since the first implementation of these chemical controls, a growing number of Irish beekeepers have been interested in adopting more sustainable practices, and numerous local reports now describe a rising number of beekeepers successfully managing colonies in the absence of treatments for multiple years, as seen in other countries [13,14,15].

A further serious impact on *A. m. mellifera* in Ireland is the increased genetic introgression deriving from the introduction of non-native subspecies from mainland Europe [16]. This phenomenon may cause the irrevocable loss of genotypes that are locally adapted to Ireland’s harsh weather, which in turn will reduce the number of efficient pollinators within the island and severely impact its ecology and food systems. Recent research suggests that *A. m. mellifera* is still common and widespread in Ireland, as are free-living colonies of this subspecies [10,17]. However, this scenario is more likely to change given the recent increase in the number of importations of non-native honey bee queens (McCann and McCormack, in press), which have likely been driven by the growing demand of local beekeepers for acquiring varroa-resistant and productive bees.

In order to resolve the barrage of issues that Irish honey bee colonies are facing, local beekeepers must be included in plans fostering national coordination and concerted actions. As such, fostering large-scale implementation of sustainable and efficient *V. destructor* control strategies, like the breeding and subsequent national distribution of local varroa-resistant bees, may be key for helping to solve these issues. Such objectives are supported by local scientific research, which aims to guide the implementation of a national breeding programme capable of fulfilling stakeholders’ needs and protecting Irish honey bees. However, no data are currently available for informing such decisions, as no study has investigated Irish beekeepers’ demographics in detail or assessed their opinions towards selection for varroa resistance or the conservation of local honey bee subspecies.

By deriving from the surveying methodology of Guichard et al. [18], we sought to assess Irish beekeepers’ demographics as well as investigate levels of interest in the selection for varroa tolerance/resistance traits and use of the local honey bee subspecies in order to understand whether a research strategy in this area would be supported by the local stakeholders. Given the rising concern around the conservation of the local honey bee subspecies, we also focused on understanding to what extent the selection of various traits is valued compared to the importance of retaining local native honey bees. We considered that applying the methodology carried out by Guichard et al. [18] would yield important insights that will be useful in directing future research planning in support of Irish beekeeping.

## 2. Materials and Methods

The survey was based on Guichard et al. [18], with slight adaptions to fit an Irish context (Table S1). The survey was divided into three parts depending on the types of questions asked (see below). Answers to questions were in a variety of formats, including user inputs (categorical or numerical), checkboxes, or text boxes. For dissemination, the survey was printed in FIBKA and NIHBS beekeeping magazines, where the link to the online version was also provided. The link to the survey was also posted on the websites of these same associations and on all the beekeeping-related Facebook pages hosted in Ireland. The survey was launched on 1 October 2020 and left open for four months. The data were subsequently collated (from hardcopy, email, and online responses), and the questionnaire and all data produced were managed following GDPR guidelines.

*Beekeepers and apiaries:* The first section of the survey was focused on understanding the experience and current apiary size of respondents. This section also contained questions related to the type of honey bee preferred by beekeepers. Respondents were also asked if they adhere to the NIHBS initiative, if they keep dark bees, and if so, why.

*Importance of breeding traits and grouping beekeepers:* This section asked beekeepers to score, from 1 (not important) to 5 (very important), 9 traits that are often sought via breeding, i.e., honey yield, gentleness, calmness on the comb, low swarming behaviour, brood health, varroa resistance, pure race, or genetic diversity. This was carried out to explore whether varroa resistance is a priority for beekeepers and if it is considered more important than other traits for which to select. Pairwise t-tests were used to understand any significant differences in the mean level of importance assigned by beekeepers to the various breeding goals. The answers provided for this section were used to cluster Irish beekeepers into groups based on the importance placed on breeding criteria. To obtain such groups, the Euclidian distance between each responder’s answers was calculated, and hierarchical clustering was carried out on the resulting distance matrix using the ward. D2 method [19]. To visualise and describe how groups differ in their responses, a heatmap was created using the mean score given to the breeding criteria by each group. Moreover, to test whether these groups differed specifically in the level of importance attributed to varroa resistance, an ANOVA test was run on the scores given to this trait by each group. Lastly, an ANOVA analysis was also run to assess whether groups differed in the average number of colonies possessed by the beekeepers composing it.

*Selective Breeding for Varroa Resistance:* Commercial beekeeping is uncommon in Ireland, with most beekeepers maintaining low numbers of colonies. Despite this, beekeepers spend significant effort in treating varroa and in determining the best methods of control. Therefore, we sought to investigate the likely decision-making of beekeepers on investing in a varroa resistance strain if it came at a cost to local bees. This explores more deeply the importance of conservation and genetic diversity to Irish beekeepers but also the relative value of honey production over colony health and how much selective force they think is optimal in a future breeding programme, if research should be largely focused in this area in the future, and if they would be willing to participate in such research. Whether beekeepers saw a use for a genetic testing facility was also asked. The responses to these questions were further assessed based on the beekeeper groups defined earlier in the clustering step.

## 3. Results

**Figure 1:**
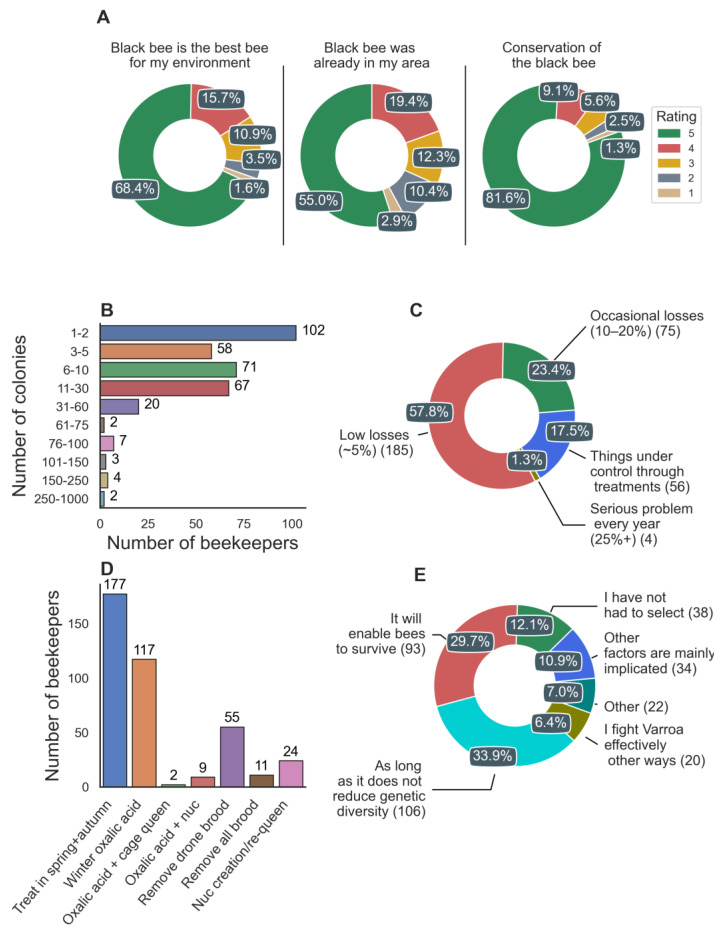
Plots of survey results. (**A**) revealing details of main reasons why respondents chose to keep the black bee, (**B**) the numbers of colonies kept by respondents, (**C**) assumed impact of varroa on colony losses by respondents, (**D**) what treatments respondents apply for varroa control and (**E**) respondents’ views on whether selection could be the solution to varroa. Numbers between brackets indicate the number of responses per category.

*Beekeepers and their bees:* The survey was completed by 342 respondents (1 via email, 15 via hardcopy, and 326 via the online version, Table S2) who own a combined total of 6274 *A. mellifera* colonies in Ireland. At the time of answering, 43% (N = 149) of the respondents were registered as NIHBS members, while 56% were not (N = 192). Over 90% (N = 310) of respondents consider that they are keeping the dark bee, and just under 6% (N = 20) state that they are not. The remaining respondents (N = 12) did not know which type of bees they kept. Beekeepers were asked to score three possible reasons for keeping the dark bee. The most important determining factor as to why Irish beekeepers keep the black bee was conservation reasons, with 81.6% of respondents giving it the highest rating of 5 (Figure 1A). The second most important factor was the view that the black bee was best for the local environment; 68.4% of respondents gave this the highest rating, while 55% thought that the simple fact of the subspecies already being here was equally of the highest importance.

Most of the beekeepers who responded to the survey did not keep honey bees for commercial purposes. Fewer than 10 survey respondents had a colony count greater than 100, with the median colony count being 6 (Figure 1B). The level of beekeeping experience ranged from 0 to 60+ years, with 5 years or less being the most selected answer (N = 156) and 5–10 years being the next largest category (N = 94). Notably, one respondent had over 60 years of experience, and two had between 50 and 59 years. Where and how beekeepers obtain their bees is an important consideration, and while most beekeepers reported a mixture of origins for their colonies, the majority responded that they were obtaining queens within their own apiary (N = 168) or area (N = 188), with a combination of these two answers being the most common. Less than 4% (N = 15) of respondents reported using a designated mating station to mate or acquire their queens. Irish breeders supply 25% (N = 85) of our respondents’ apiaries with queens, while just 2.5% (N = 8) bought queens from a breeder operating out of another country.

*Varroa control:* We explored how much of a problem varroa is for Irish beekeepers. Only 1.3% (N = 4) of respondents found varroa to be a serious problem every year, while around a quarter have yearly colony losses in the region of 10–20% that they can attribute to the mite (Figure 1C). The majority (75.35%, N = 185) could only attribute ≤5% of yearly colony losses to varroa infestation. To assess whether efforts to keep losses at such a low level occupy a substantial amount of time for beekeepers, we asked how often they inspect for varroa and which treatments they employ. Varroa inspections are carried out in some form by 87.5% (N = 290) of respondents, of which 64% (N = 204) also employ record-keeping practices of these treatments. However, 22% (N = 75) of respondents claim that they do not treat for varroa. The treatment strategy that respondents use for varroa control varies, but the majority (51%, N = 177) are using chemical treatments (i.e., oxalic acid) in spring/autumn and winter (Figure 1D). There is a subset of beekeepers (26%, N = 90) who use biomechanical methods for varroa control, such as removing drone/all brood and frequent nucs creation. Notably, these beekeepers reported similar losses compared to those who applied chemical treatments.

*Artificial selection and honey bee traits*: When asked whether selection should be carried out on the black bee as a way of improving overall bee stocks on the island, the majority of beekeepers believe that it should be carried out in some form (Figure 1E), with the answers split between total selection (e.g., eradication of poorly performing queens) and partial selection (e.g., breeding the best queens but allowing the poorly performing ones to continue naturally). Many also responded that selection should only be carried out as long as it did not reduce the genetic diversity of honey bees in Ireland (Figure 1E). Other factors (environment, beekeeping practices, etc.) were recognised as more important than selection to the survival of the black bee by approximately 17% of respondents (Figure 1E).

**Figure 2:**
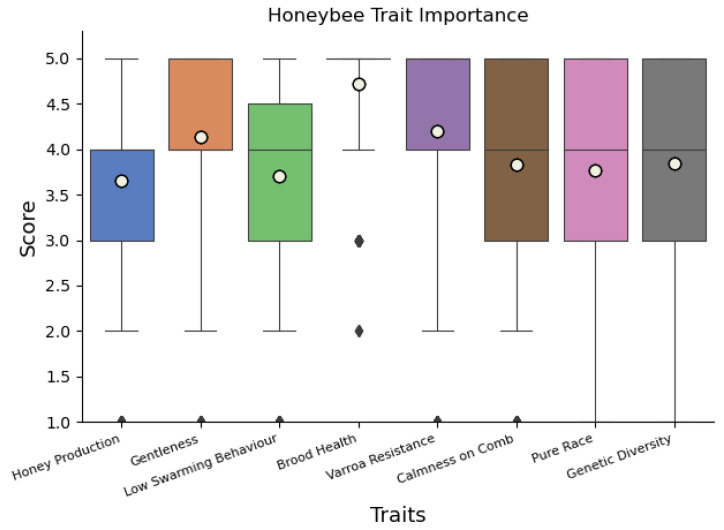
Boxplot of scores given to each breeding criteria, where a score of five is most important. Mean value for each trait is displayed as white dots. Black lines within each box represent medians. Top and bottom of each box represent the interquartile range of the responses (Q3, Q1), while the whiskers represent the 0.05 to 0.95 range of values. Outlier values outside of this are marked with a grey diamond. All traits except brood health and varroa resistance were given a median score of four.

When beekeepers scored eight traits from 1 (not so important) to 5 (very important), all traits were deemed to be important, but brood health was scored significantly higher in comparison to all other traits (Figure 2, Table S3).

**Figure 3:**
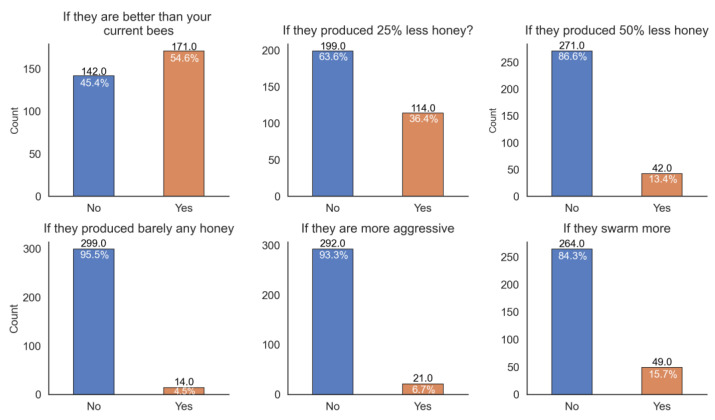
Responses of beekeepers relating to their willingness to swap to a new varroa-resistant line under different conditions, e.g., a varroa-resistant strain with lower honey yield, higher swarming behaviour, or aggressiveness.

To investigate whether Irish beekeepers regard the use of local and native honey bees as more important than a possible improvement in breeding criteria or traits, we asked a series of questions related to swapping to a newly developed line of honey bees. When asked if they would swap from their current stock to a varroa-resistant line of *A. m. mellifera* bred in Ireland, 54.6% of respondents would swap to resistant bees if the resistant line was better than their current one (N = 171, Figure 3). As shown in Figure 3, traits such as honey yield, gentleness, and swarming behaviour in people’s own bees are important to most respondents. Notably, however, some responded that they would swap even if the resistant bees only produced up to half the normal amount of honey or had a higher swarming tendency (N = 42, N = 49, respectively, Figure 3). An extremely low proportion (6%, N = 21) of respondents would swap to resistant bees if they were more aggressive than their current honey bees, suggesting that gentleness is an especially important factor for Irish beekeepers.

When asked about the level of focus that should be placed on developing varroa resistance in honey bees versus other traits of interest, 17.6% (N = 54) responded that all focus should be placed on varroa resistance. The majority, 60.5%, still thought that varroa resistance is a main goal but that 50% of the focus should be placed on other traits, and just over a fifth of respondents considered that 80% of the focus should be spent improving these other areas. Overall, almost 85% (N = 269) of respondents answered yes to the question asking if they thought bee-related research should work towards long-term varroa resistance in *A. m. mellifera,* with over 80% of respondents willing to participate in such research. The reasons given as to why beekeepers would not want to participate in research or have their honey bees genotyped ranged from their own perceived lack of experience to a general distrust of scientific interference.

**Figure 4:**
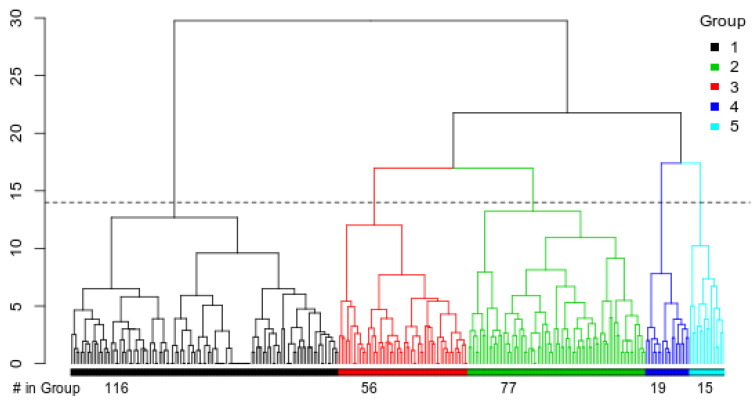
Grouping of beekeepers by importance placed on various breeding criteria using a Euclidean distance matrix and Ward.D2 hierarchical clustering. Only respondents who supplied a score for every trait were included. Beekeepers clustered into five distinct groups.

*Beekeeper traits:* The finding that there are significant differences between the breeding criteria of Irish beekeepers who responded to the survey raises the question of whether there are distinct groups of beekeepers operating in Ireland. To investigate this, hierarchical clustering using Euclidean distances was performed on the answers that were given to the breeding criteria questions. Beekeepers were clustered into five groups, as shown in Figure 4. Of the five groups of beekeepers, the largest cluster, Group 1, was more distinct from the others, while the smallest groups, 4 and 5, are more similar to each other than to the intermediate groups, 2 and 3 (Figure 4). Notably, no significant difference was found in the average number of colonies possessed by beekeepers of the various groups (*p*-value = 0.30309).

Beekeepers in Group 1 rated all criteria, except honey production, as very high (4+) (Figure S1). Beekeepers of Group 2 placed high importance on traits associated with a healthy natural colony (e.g., brood health) and lower importance on traits related to commercial needs (respectively, low swarming, honey production, and calmness on comb). Group 3 considered genetic diversity as the least-important criterion; Group 4 placed low levels of importance on the purity of race (1.11), while beekeepers in Group 5 placed low importance on both genetic diversity and purity of race (1.13 and 1.27). Regarding varroa resistance, there was a statistically significant difference in the importance of this trait between the groups (*p*-value = 1.5 × 10^−24^), with beekeepers in Groups 1, 2, and 3 rating it as high importance and Groups 4 and 5 as low importance. Notably, most beekeepers in Groups 4 and 5 (71% and 78%) reported that *V. destructor* was not a problem for them in that they experienced only approximately 5% of colony losses due strictly to this parasite. By comparison, fewer beekeepers in Groups 1, 2, and 3 considered varroa not to be a problem (54%, 57%, 49%). In line with this, fewer beekeepers in Groups 4 and 5 agreed that research work on long-term varroa resistance was important (64% and 56%) compared to Groups 1, 2 and 3 (94%, 84%, and 91% agreeable). The number of beekeepers in each group is supplied in Figure 4.

## 4. Discussion

By following an established methodology [18], we collected data on the opinions and perceptions of Irish beekeepers to inform the future development of a breeding programme at a national level. We obtained a coverage similar to that of previous surveys that included Irish beekeepers [20], as well as studies with similar scopes conducted at a national scale [21]. Our results confirm that beekeeping in Ireland is mainly practised at a hobbyist level [8], with the majority of beekeepers managing fewer than 30 colonies. Furthermore, most respondents reported keeping the native honey bee subspecies, with a keen interest in conserving native biodiversity. Such factors will be important to incorporate into any future Island-wide sustainable beekeeping strategy, as beneficial results in the breeding of varroa-resistant lines are only likely to be achieved if strategic decisions consider stakeholders’ opinions and preferences.

Considering the responses gathered here on beekeepers’ opinions towards a future breeding programme, we consider our survey to have obtained a good representation of the portion of Irish beekeepers who are opinionated and interested in improving bee breeding in Ireland. Moreover, the methodology applied enabled the profiling of Irish beekeepers based on their preference over various breeding criteria and their subsequent clustering into different groups. Our objective was to understand the different opinions that are prevalent among the community and to cater to each group’s needs in the future, should a sustainable breeding programme be established. The largest group (Group 1) rated all traits except honey production as highly important. This group represents beekeepers who look for bees that excel in all areas. While some breeding initiatives have already obtained positive results in the simultaneous improvement of multiple traits [22], such projects may be difficult to implement in Ireland, where no supporting infrastructure for bee breeding is currently available. At present, the results are probably more attainable if the selection is optimised for fewer traits that are less likely to be influenced by the environment [23]. The second-most abundant cluster (Group 2) describes beekeepers who want to keep bees as a hobby by making sure they are healthy and not hybridised but allowing them to be wild-like. For example, they see swarming in a more positive way than any of the other groups. This group can be viewed as the group which is most focused on the conservation of the Irish honey bee, allowing it a place to dwell and treating it for disease as needed. Group 3 seeks to breed healthy bees but is not too concerned about the genetic diversity of their honey bees. Genetic diversity can be a key indicator of the health of a population, so this is at odds with their goals [16,24]. They want varroa-resistant honey bees that are also calm, gentle, and easy to work with. The second least-abundant cluster (Group 4) instead desires honey bees that are healthy, produce a lot of honey, and are very genetically diverse. They also rate low swarming behaviour and gentleness as of high importance. They do not care if their honey bees are hybridised or if they are varroa-resistant. These characteristics would seem to describe a beekeeper who is mostly interested in honey bees that are easy to manage and will create some return on their investment. Lastly, the smallest group (Group 5) places low importance on most of the traits, and they particularly do not care about the hybridisation levels of their honeybees or their genetic diversity. Their profile shows a mild interest in breeding colonies that have high honey production but rate all other traits as low importance, except for brood health. This group would most represent those beekeepers who do not care about the conservation of any type of subspecies and are simply happy to have healthy colonies in their apiaries. Whether this is due to an unfamiliarity with the concept of conservation and how genetics play a role in the health of a species or if it is based on a lower attractivity of the native subspecies in comparison to other lineages or hybrids is unknown.

Although variable preferences towards breeding criteria were identified within the groups, most Irish beekeepers see selection as the solution to varroa. This common agreement was expected given the impact of this parasite on honey bee health and beekeeping at large [12], and it should be viewed positively, as it is likely to align with Irish beekeepers’ interest in honey bee breeding, an aspect that would increase the likelihood of success of future nation-wide selection plans. However, only a very small fraction of respondents considered this parasite to be a serious threat to their colonies, while the majority attributed very low annual colony losses to its infestation (i.e., ~5%). These statements may appear surprising since it would be expected that the strong interest of Irish beekeepers towards the selection for varroa resistance would emerge in circumstances where such a parasite is causing high mortalities and is perceived as a threat. However, while the apparent low varroa-related losses reported may be explained by the efficacy of the chemical treatments applied by beekeepers [25], their general interest in selecting against this parasite may be rooted in the intention of promoting practices allowing for a better beekeeping experience. Notably, this scenario presents strong parallels with what has been found for UK beekeepers, where a similar proportion admitted to not treating against the parasite [21]. Since a substantial proportion of a beekeeper’s time and money is invested in treating against varroa, selection for resistance will promote beekeeping free of these concerns. Alternatively, a possible explanation for the apparent low concern of Irish beekeepers towards this parasite may be ascribed to the potential expression of resistance in Irish honeybees, which would enable colonies to control the parasite’s infestation on their own [26,27], or the presence of particular adaptations in the parasites [28,29]. However, even though such a possibility has been stated before [30,31], experimental evidence confirming such hypotheses is still missing for Irish honeybees. By taking advantage of established investigation frameworks available for the study of host resistance in *A. mellifera* [30,32], future research could thus focus on investigating the colonies kept by the portion of ‘treatment-free’ beekeepers (16% of the total respondents) that in our survey reported low varroa-related colony losses.

The characteristics of the starting population are crucial for determining the success of any selection programme [23]. Furthermore, studies on genotype-per-environment interactions demonstrated that local colonies outcompeted non-local ones in terms of survivability and resistance [33,34]. As such, any future nationwide breeding plan will benefit from founding stocks of local origin, and, as 90% of respondents here reported keeping the native black bee, it appears that there is a good base from which a breeding programme could be successfully initiated in Ireland. In addition, over 70% of the beekeepers stated that they would be interested in swapping to a varroa-resistant line derived from local honey bees, and very few respondents admitted having acquired queens from breeders abroad. We thus have reason to believe that once a local breeding programme starts producing queens born of locally resistant stocks, their uptake within the local community will be swift. Altogether, these results are very encouraging for the successful implementation and maintenance of a future nationwide selection programme in Ireland.

On the other hand, the preferences of the remaining small proportion of beekeepers who expressed interest in acquiring resistant queens, even if produced from non-native *A. m. mellifera* or from other subspecies, should also be considered. Since traits related to resistance against *V. destructor* have been identified in other non-Irish *A. m. mellifera* [27], while a good number of hybridised stocks are commercialised as resistant in Europe, there is the possibility of an increase in the introduction of non-native lineages within the island. This may result in hybridisation of the local line and could lead to damaging future effects, such as loss of genes that are specifically adapted to the local environment [10,16,35]. Moreover, if future demand increases past local supply, the possibility of beekeepers seeking queens from abroad may also increase, even among those who prefer local bees. A breeding programme must therefore plan for this possibility and have local queens, which have been selected for the trait of interest, ready to supply new beekeepers so as to keep the area insulated from outside sources of genetic variation. Such a result could be achieved by investing in the education of local stakeholders towards bee breeding practices for conservation, as recently initiated by the NIHBS queen rearing group scheme (nihbs.org, accessed on 19 October 2023). Notably, such a strategy may prove to be particularly efficient if directed towards those beekeepers constituting Groups 1 and 2, who may find such training to be in line with their personal interests, given the importance they placed on the improvement in multiple breeding criteria and/or the conservation of the local bee.

It appears that the desirability of Irish beekeepers for a breeding programme is not only motivated by the interest in obtaining varroa-resistant bees but also more productive and manageable ones. The answers we collected suggest that the majority are keen to acquire a new line of bees only if such a line is as productive or as gentle as their current one. This is in contrast with what was found in Swiss beekeepers answering similar questions [18] and may be difficult to attain, as resistant colonies could have lower honey yield and may express traits less fitting to modern beekeeping (i.e., low gentleness, increased swarming) than susceptible ones [36,37]. As such, before the initiation of a breeding programme in Ireland, there may be a need to mediate beekeepers’ expectations and better understand their level of commitment in case colonies with less-interesting economic traits are produced.

Finally, among the respondents, there is a high predominance of beekeepers stating that they keep colonies of the local black bee and have an association with the NIHBS. This appears to be motivated by the intention of supporting and conserving the local environment. This high proportion and positive intentions are highly encouraging in light of the current national initiative to protect the local black bee [9]. It is therefore in the interest of Irish beekeeping and conservation attempts that breeding for varroa resistance should be given a substantial focus in Ireland. Irish beekeepers are in agreement with this, with 78% of respondents believing that at least 50% of local scientific research efforts’ focus should be on varroa resistance. However, through our clustering analysis, it also appeared that the smallest groups, 4 and 5, reported lower concerns regarding varroa in their colonies and were less in favour of scientific research focusing on resistance. These two groups were also the least convinced that selection is a way to enable honey bees to survive varroa. Their participation in a scientific breeding programme focussed solely on varroa resistance is, therefore, unlikely to occur unless other possible outcomes fitting to their needs are also explored (e.g., diversification of income through queen production). Therefore, the most probable source of beekeepers for a *Varroa* resistance breeding programme would be beekeepers that fall into the profiles of the other three groups identified in this study.

## 5. Conclusions

The majority of beekeepers who responded to the survey are hobbyists in nature and keep the native subspecies, *A. m. mellifera*. Most beekeepers inspected and treated their colonies against varroa. However, a reasonable number reported not treating their colonies and observing similarly low levels of impact due to this parasitic mite to those who do treat. Brood health and varroa resistance were two of the most sought-after traits, and most respondents would be interested in switching to a varroa-resistant line of honey bees, provided they were also productive and calm. Most beekeepers would retain their own local bees if the varroa-resistant line were not of the native subspecies or not of Irish origin, although some beekeepers were suitably interested in such traits that they would bring in such lines of bees from outside. There is a clear appetite for research to focus on the breeding and selection of native local honeybees for varroa-resistance traits in Ireland, and beekeepers appear to be motivated to partake in it.

## Data Availability

Data used for the analyses are available in Table S2.

## Supplementary Materials

The following supporting information can be downloaded at: https://www.mdpi.com/article/10.3390/insects14100827/s1, Table S1: List of survey questions. Table S2: Anonymised answers to the survey. Table S3: Comparisons between the mean scores given to each breeding criterion. Figure S1: Heatmap of the mean importance placed on breeding criteria.
