# Supplementary material for: Exploring a Potential Avenue for Beekeeping in Ireland: Safeguarding Locally Adapted Honeybees for Breeding Varroa-Resistant Lines
Source: Insects. 2023 Oct 20;14(10):827. doi: 10.3390/insects14100827 (PMC10607453; doi:10.3390/insects14100827)
Supplement: Supplementary file 1 [file insects-14-00827-s001.zip › Figure S1.pdf]

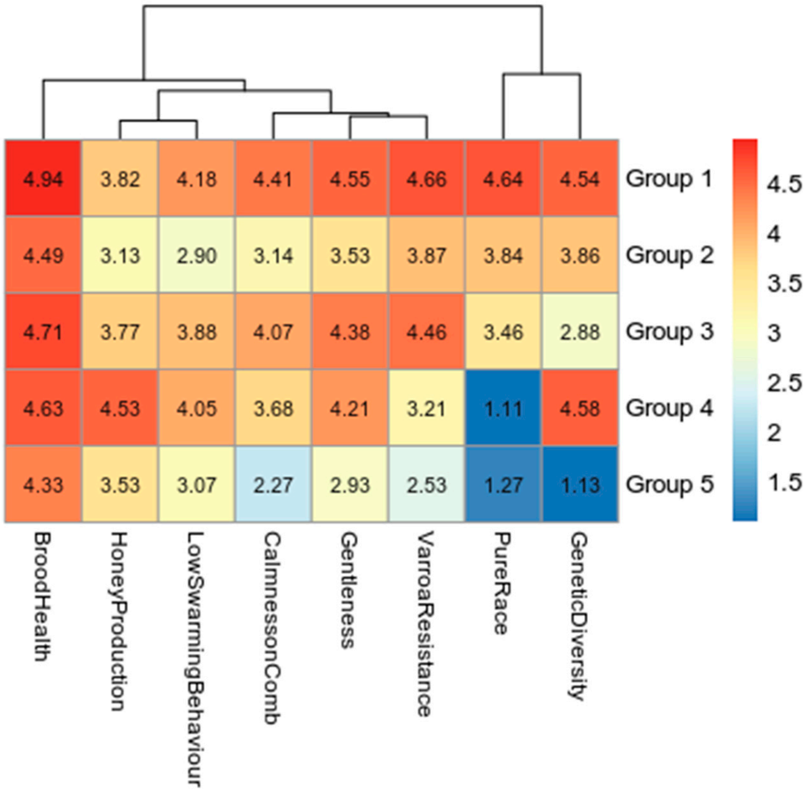

Figure S1. Heatmap of the mean importance placed on each breeding criteria by the groups of beekeepers with the traits clustered using the Canberra distance. Genetic diversity and purity are the traits which differ the most between groups.
