# Supplementary material for: Exploring a Potential Avenue for Beekeeping in Ireland: Safeguarding Locally Adapted Honeybees for Breeding Varroa-Resistant Lines
Source: Insects. 2023 Oct 20;14(10):827. doi: 10.3390/insects14100827 (PMC10607453; doi:10.3390/insects14100827)
Supplement: Supplementary file 1 [file insects-14-00827-s001.zip › Table S3.pdf]

Table S3. Comparisons between the mean scores given to each breeding criteria trait by Irish beekeepers. Values below the diagonal are p-values resulting from pairwise t-tests. Values above the diagonal indicate the differences in mean scores between each pairing. The mean score given to brood health is significantly higher than that of all other traits.

Trait	Honey Production	Gentleness	Low Swarming Behaviour	Brood Health	Varroa Resistance	Calmness on Comb	Pure Race	Genetic Diversity
**Honey Production**	-	-0.477	-0.05	-1.067	-0.544	-0.18	-0.12	-0.195
**Gentleness**	3.117E-08	-	0.427	-0.59	-0.067	0.297	0.357	0.282
**Low Swarming Behaviour**	0.5727	4.686E-07	-	-1.017	-0.494	-0.13	-0.07	-0.145
**Brood Health**	2.2E-16	2.2E-16	2.2E-16	-	0.523	0.887	0.947	0.872
**Varroa Resistance**	6.316E-10	0.4169	1.15E-08	8.99E-14	-	0.364	0.424	0.349
**Calmness on Comb**	0.04156	0.00047	0.1342	2.2E-16	2.70E-05	-	0.06	-0.015
**Pure Race**	0.2392	0.00033	0.4845	2.2E-16	2.26E-05	0.5543	-	-0.075
**Genetic Diversity**	0.04045	0.002	0.1223	2.2E-16	0.00017	0.8807	0.488	-
